# Women’s visibility and bargaining power in the common bean value chain in Mozambique

**DOI:** 10.1186/s43170-023-00197-9

**Published:** 2023-11-30

**Authors:** Enock K. Maereka, Eileen B. Nchanji, Victor Nyamolo, Lutomia K. Cosmas, Bartholomew Y. Chataika

**Affiliations:** 1 International Center for Tropical Agriculture (CIAT), Chitedze Research Station, 15Km Mchinji Road, P.O. Box 158, Lilongwe, Malawi; 2 CIAT, Duduville Campus, Off Kasarani Road, P.O. Box 823-00621, Nairobi, Kenya; 3 University of Egerton, Nakuru-Mau Narok Road, P.O. Box 536 - 20115, Egerton-Njoro, Kenya

**Keywords:** Gender, Intersectionality, Food security, Income, Common bean

## Abstract

Women are involved in bean production and marketing, but their contribution is often invisible. This study is interested in understanding gender gaps in bean production, marketing, and decision-making powers over income and sales. A mixed method was used to collect survey data from 332 farming households and qualitative data from focus group discussions and key informant interviews. The respondents in the study were young men (30.42%), young women (13.25%), adult men (35.84%) and adult women (20.48%). From the results, adult men and young men owned more land than adult women and young women. Common bean had the highest median price of MZN25.00 (US$1.25) per kg over other crops such as maize at MZN7.00 (US$0.35), soybean at MZN 10 (US$0.50), groundnut at MZN 17 (US$0.85) and cowpea at MZN10.00 (US$0.50). The study revealed gender disparities in the control of bean sales and income. While there were no differences in the volume of bean grain sales between adult men and adult women, there were significant differences (p < 0.05) between young men and young women. Compared to young women, young men sold twice the bean grain volume and earned nearly twice more income, despite the two groups producing comparable volumes of bean grain. In conclusion, unlike the prevailing consensus that legumes are women’s crops, the economic benefits, particularly in common bean, accrue more to young men than to any other category in this study.

## Introduction

In the year 2022, Mozambique celebrated 30 years of the end of a major civil war that ravaged the country for 15 years. These celebrations are a time to reflect on whether the gains of independence and peace have been consolidated and transformed into improving agricultural productivity and food security, poverty reduction, and meeting the Sustainable Development Goals (SDGs). In 2021, Mozambique was ranked 185 out of 191countries on the human development index (HDI) rank (HDR 2021/ 2022), with 72.5% of its population living in multi-dimensional poverty, and 62.4% of the population living below the purchasing power parity income poverty line of US$1.90. Social and economic inequalities still undermine development in Mozambique despite growing global calls for addressing pervasive historical injustices and inequalities (Miller and Davis-Howard 2022). Women have been disadvantaged through various cultural norms, resulting in lower earnings, less wealth accumulation, and less participation in corporate leadership than men (Chancel et al. 2021). This comes against the backdrop of recent evidence showing that women significantly contribute to various sectors of the global economy (Nguyen 2021). Thus, addressing gender inequalities in Mozambique requires understanding the root causes to help co-develop activities that can transform the economy. Mozambique’s economy is buttressed by agriculture, which employs about 70% of the country’s active workforce (FAO 2020), of which 59% is female labour force, the second highest in the world after Nepal (FAO 2021). In recognition of this, the government of Mozambique has strategic plans and programs aimed at accelerated inclusive agricultural growth for improved food and nutritional security, farm income, and poverty reduction among poor households in rural areas. Among the strategies is the promotion of the production of grain legumes, including the common bean (*Phaseolus vulgaris*), for consumption, income generation, and resilience to climate change crises.

### Gender gaps in agriculture

Within the agriculture sector, there are gender gaps across the value chain, especially in technology adoption (Mugisha et al. 2019), productivity (Muricho et al. 2020), resource endowment and control (Huyer 2017), and skills and knowledge among many others. While the contribution of women to agriculture in Africa cannot be overlooked, studies within the region have reported that the gender gap in agriculture costs not only the women but the entire countries of Malawi, Tanzania and Uganda a combined total of US$270 million per year in lost productivity (UN-Women et al. 2015). In particular, data from surveys in Mozambique reported a 20% agricultural productivity gap between men and women farmers due to gender inequalities in resource endowment and technical efficiency (Morgado and Salvucci 2016). Addressing these gender inequalities requires a clear understanding of their nature and scope, which vary across value chains (production, distribution and consumption hubs), therefore requiring contextual strategies.

Diversity, equity, and inclusion (DEI) policies (Hoang et al. 2022; Lyman et al. 2022) are in the spotlight in contemporary workplaces and economic sectors, and correspondingly, the inclusion and empowerment of women and youth in agriculture has gathered pace in recent times. Practitioners in agriculture and rural development have also lined up strategies to recognize and enhance opportunities for women and other previously disadvantaged and minority groups. Numerous past studies have highlighted the participation of women in agriculture while very little information about the youth is available. Similar to women, the youth have also been sidelined in economic development (Bank 2006), yet they will soon make up 50% of the global population, and their proportion is rapidly growing, especially in Sub-Saharan Africa (UN-DESA 2022; Vollset et al. 2020). Therefore, transforming the landscape to benefit these previously disadvantaged groups requires deliberate long-term designs, plans, activities and metrics (Johnson et al. 2018; Tavenner and Crane 2022) and a clear understanding of the *status quo*.

This paper elucidates the nature and scope of participation and bargaining power of women and youth in the common bean value chain in Mozambique. We framed the following three questions; (1) What is the place of women and youth in the bean value chain? (2) Who has access and control over resources in common bean production? and (3) Are there gendered points of sale and bargaining power in the value chain?

## Theoretical framework

This study was framed around intersectionality which recognizes the existence of multiple ways in which gender and different forms of social inequality intersect and shape people's experiences and opportunities. Intersectionality suggests that women, men and youth experiences and opportunities can be shaped by a complex interplay of factors including gender, age, education, marital status, social class, social norms, wealth, experience, geography and others.

Gender is one of the most significant factors that shape women's experiences in the common bean value chains in Mozambique. As highlighted by (Makate et al. 2018), gender plays a determining role in several farming decisions in smallholder farming. Women farmers often face limited power and control over resources compared to men, which can restrict their involvement in crucial decision-making processes related to various agricultural production and marketing practices. Despite being relatively responsible for agricultural work, including the production and processing of common beans in the Angonia district of Mozambique, women farmers are frequently excluded from decision-making processes and have limited access to resources such as land, credit, and markets (Makate et al. 2018). Thus, significantly limiting their ability to fully participate in the value chains and benefit from their involvement.

Ethnicity and social class are also critical factors that determine women’s participation in the bean value chain. Ethnicity plays an important role in shaping women's experiences in common bean value chains in Mozambique. Women from minority ethnic groups face significant barriers to accessing resources and opportunities due to discrimination and stereotypes. (Giroud and Huaman 2019) found that women from minority ethnic groups often face different kinds of discrimination and marginalization that limit their access to resources and opportunities. Moreover, they are also subjected to stereotypes and biases that affect how they are perceived by other actors in the value chain.

In addition to ethnicity, social class is another major factor that shapes women's experiences in the common bean value chain in Mozambique (Ngepah 2019). Women from lower socio-economic backgrounds have fewer access and control over resources and opportunities, making it more difficult for them to participate fully in value chains and to benefit from their participation. They may also be subject to exploitation and abuse by more powerful actors in the value chain.

Finally, social norms are another factor that influences the experiences of women in the common bean value chains in Mozambique. Women in rural areas face greater challenges in accessing markets thus limiting their ability to fully benefit from bean value chains (Chagomoka et al. 2014; Ngepah 2019). Additionally, they may be subject to more conservative social norms and expectations that limit their mobility and market opportunities.

Young men and women often suffer from limited land access, lack of knowledge and skills, limited finance, and inadequate infrastructure. Addressing these gaps requires land reform, capacity building, access to finance, infrastructure development, and mentorship programs.

Being married or not as well as being part of a male or female headed households also affects access and control over resources and decision-making power within diverse households. Even though women in male headed households sometimes have access to more land than unmarried women, many studies have shown that unmarried women or women in female headed households make more decisions and are more empowered (KNBS et al. 2015).

By adopting an intersectional approach we are able to unearth women's visibility in common bean value chains in Mozambique, and empower young farmers to participate, enhance food security, and improve their economic prospects. Understanding the complex and intersecting factors that shape their experiences and we can co-develop targeted interventions towards more inclusive and equitable value chains that benefit women and youths.

## Methodology and study area

### Study area

**Fig. 1:**
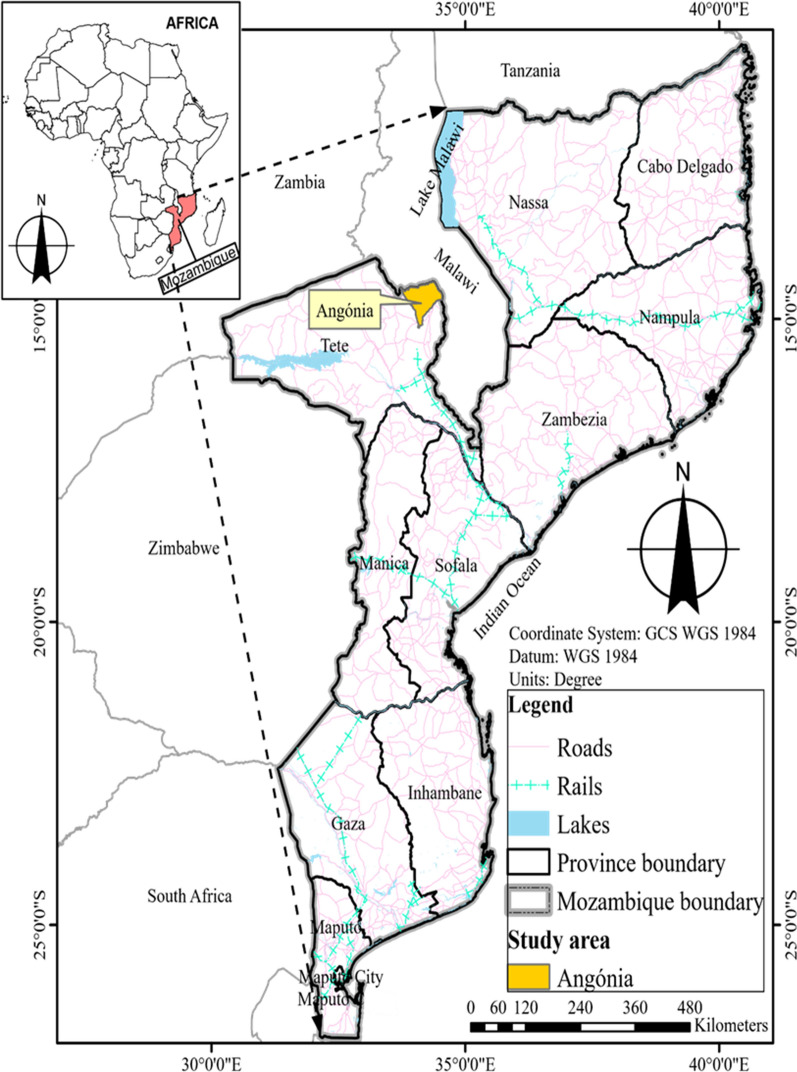
Map of study area

The study was carried out in Angonia district which is situated in the northern part of Tete Province in the central region of Mozambique, bordering Malawi (Fig. 1). The district of Angonia offers an exciting scenario among the other bean-producing districts in the country. It is the breadbasket to the sprawling and semi-arid Tete urban area. Specifically, known for its diverse bean types, Angonia attracts bean grain traders from as far afield as Maputo, which is nearly 2000 km away.

With the increasing market opportunities in Tete and its surroundings due to the expanding mining sector, small-scale irrigation could help farmers to produce high-value agricultural products and improve their capacity to respond to emerging demands in the market. Angonia district also has a high population density, which has significantly reduced the land holding per family, hence irrigation becomes critical for intensification. Angonia district is relatively rich in agricultural and animal resources with very arable land. However, the livestock is not used for land preparation, leaving most farmers to use hand hoes for land preparation activities.

### Sampling strategy

A mixed method was used to collect both quantitative and qualitative data. Quantitative data as collected from 32 *Povoado*s in Angonia district through a survey. Stratified random sampling was used following the administrative structure of the district and the respective proportional population of each administrative unit. Typically, a district in Mozambique has the following five-tier administrative structure, in descending order: *Distrito* (District), *Posto Administrativo* (Administrative Post), *Localidade* (locality/ward), *Povoado* (settlement) and *Povoação* (village). However, for this study, the smallest unit considered was the *Povoado*. Angonia District has two Administrative Posts, namely, Domue and Ulongue. The two Administrative Posts were considered the main sampling strata and we assumed that the population characteristics were uniform. The District in total consists of 18 *Localidades*, 11 in Domue and 7 in Ulongue. From these *Localidades*, it was predetermined that the sample would be drawn from eight *Localidades*. Proportionate to the number of *Localidades* in the two Administrative Posts, three *Localidades* from Ulongue and five from Domue were selected.

**Fig. 2:**
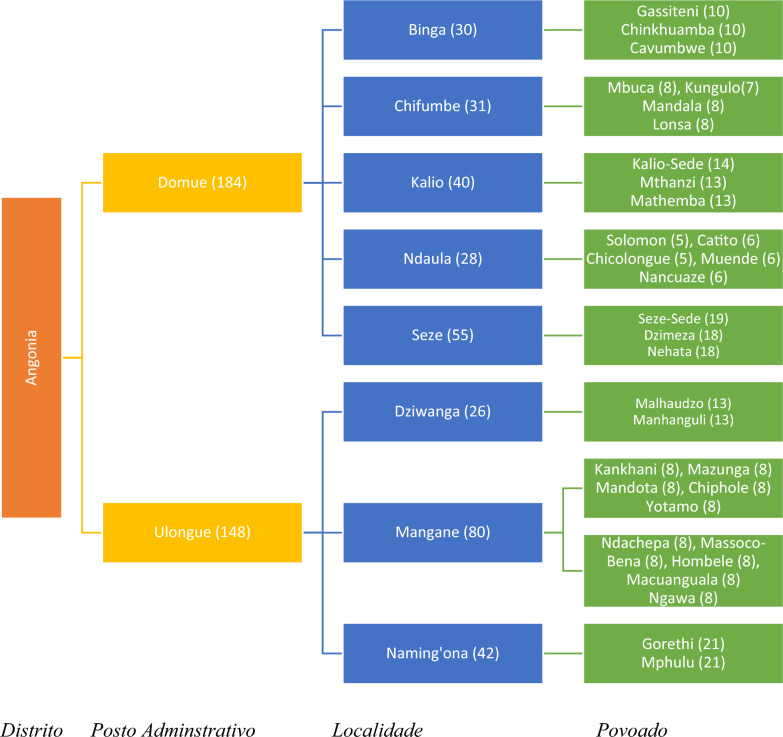
Schematic representation of the sampling frame; figures in parenthesis represent the number of households

The selected *Localidades* were then treated as the second-level strata. Using population proportions from the selected eight *Localidades* and the predetermined sample size of 332 farming households, 184 households from Domue and 148 households from Ulongue were randomly sampled. All households at the *Povoado* level had an equal chance of being selected as we applied a strict random sampling strategy with the help of local community leaders. The survey was carried out in eight *Localidades* of Angonia district. Using a list of *Povoados* in the selected *Localidades* obtained from the District Secretary’s Office and population proportions, 32 *Povoados* were thus randomly selected; 14 from Ulongue and 18 from Domue. Farming households were then selected randomly from the targeted *Povoados*. The distribution of *Povoados* and the number of households selected for the study are listed in Fig. 2.

The data were collected using a structured survey questionnaire. The questionnaire collected information on household demographic characteristics, agricultural production and marketing, adoption of improved bean varieties, income, assets and bean value chain decision-making.

The questionnaire was pre-tested and corrected for errors before administering for final data collection. Face-to-face interviews with 332 smallholder farmers were done to collect the data. We collected information on beans and other crops to establish the general agricultural crop production trends and interrelationships between the various crop enterprises then. The survey was well-timed as it was conducted soon after harvesting the 2014/15 summer crop for easy recall. Participation of respondents was voluntary. Where possible, respondents were gathered at selected points by the local leadership, but in other locations, enumerators met respondents at their households. We classified the respondents into four groups: young men and young women (35 years of age and below) and adult men and adult women (older than 35 years of age).

In addition to the questionnaire, we collected qualitative data from two focus group discussions (FGDs), one in each of the two administrative posts. The FGDs comprised representatives from all four categories of study participants: adult men (10), adult women (9), young men (11) and young women (11). The FGDs were divided into adult men and young men and also adult women and young women. We acknowledge that this might create bias as the young people might not be able to express themselves in the presence of their elders due to the existing cultural norms. Furthermore, we had eight key informant interviews (KII) with agricultural extension officers (3), farmer group leaders (3) and local traditional leaders (2).

### Data analysis

The qualitative data was recorded and transcribed word verbatim. It was later coded according to relevant themes and analysed using context analysis. For the quantitative analysis, it was performed to measure the distribution of continuous variables while the chi-square test of independence was performed to estimate the differences in the distribution of categorical variables. Means were used to describe continuous numeric variables while cross-tabulations of categorical variables were performed to obtain frequencies and percentages of farmers’ responses. The qualitative data complemented information from the quantitative study, explained the why and provided thick description.

## Results

### Socio-demographic data of respondents

**Fig. 3:**
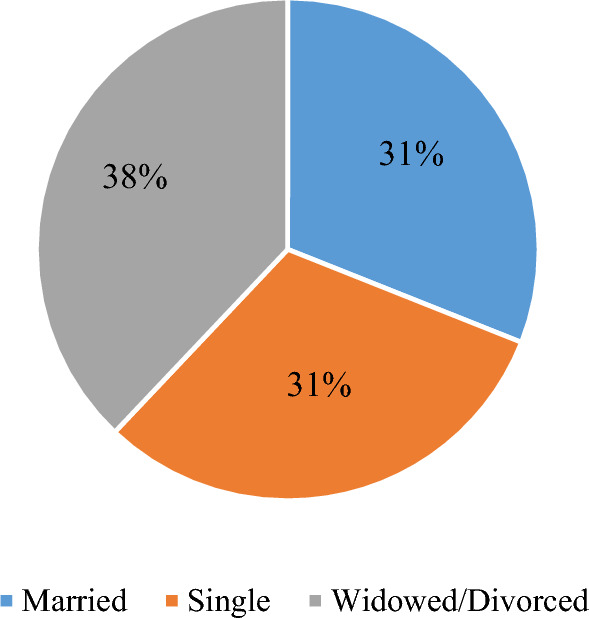
Marital status of women household heads

Table 1 presents the socio-demographic characteristics of respondents. The participants comprised 30% young^1^ men, 13% young women, 36% adult men, and 21% adult women. Overall, the average age of respondents was 40 years, while it was 28 years for the youth, and 50 and 51 years for adult women and adult men respectively. There were no significant age differences between adult men and adult women respondents in each age category. The majority of the respondents (79%) were married, while the remaining were single (16%) or divorced/widowed (5%). Seventy-nine percent of the respondents were household heads. A significantly higher proportion (p < 0.000) of adult men (more than 96%) were reported to be household heads, compared to only 45% among adult women respondents. Women household headship was rare among married women (31%) but was common (69%), only when the woman was either divorced or widowed (Fig. 3). More adult women than adult men attended the first five years of education across both age groups, but more men than women were reported to have attended the later years of education (9 years and 13 years). These differences were not statistically significant, but noteworthy is that in the younger generation, more younger women respondents reported having spent more time in school compared to the older generation. Nearly all the respondents (99.7%) reported farming to be their main occupation and there were no significant differences in the main occupation by gender.

**Table 1:** Socioeconomic characteristics of respondents by gender

Variable	Total (N = 332)	Young men (n = 101)	Young women (n = 44)	p-value	Adult men (n = 119)	Adult women (n = 68)	p-value
Gender of the respondent (%)		30.42	13.25		35.84	20.48	
Age of respondent (years)	40.2	27.81	27.89	0.927	49.76	50.62	0.588
Marital status (%)				0.522			0.000
Married	78.53	83	81.4		81.9	64.18	
Single	16.26	16	13.95		16.38	17.91	
W/D^a^	5.21	1	4.65		1.72	17.91	
Household head (%)	79.22	96.04	45.45	0.000	97.48	44.12	0.000
Education level (%)				0.327			0.092
5 years	74.48	56.94	71.43		81.18	91.49	
9 years	21.34	36.11	20.00		16.47	8.51	
13 years	4.18	6.94	8.57		2.35	0.00	
Full time farmer (%)	99.7	99.01	100	0.511	100	100	0.524
Farming experience (years)	14.93	6.25	7.93	0.079	20.61	21.88	0.517
Land owned (acres)	3.52	3.27	2.59	0.038	4.22	3.27	0.009
Land accessed (acres)	3.67	3.48	2.85	0.072	4.32	3.31	0.006
Land ownership in household				0.398			0.558
Man	29.82	34.65	18.18		39.5	13.24	
Woman	26.20	18.81	38.64		10.92	55.88	
Both Man and Woman	43.98	46.53	43.18		49.58	30.88	

^a^Widowed/Divorced

^a^During the time of the study, the exchange rate was US$1:MZN34.8

^a^*MZN* Mozambican Meticals*, MZN1 *is equivalent to US$0.05

Across the two age groups, adult women had slightly higher but non-significant bean farming experience than adult men. Young women had an average of nearly two more years of bean farming experience compared to the young men’s average of six years. In the older generation, adult women had slightly more than a year of bean farming experience than adult men.

Land ownership and access to land differed significantly by sex (p < 0.05) among both adults and youth. The size of land owned (4.22 acres) and accessed (4.32 acres) by adult male farmers was significantly larger than that owned (3.27 acres) and accessed (3.31 acres) by adult women respondents. Similar to adults, young men owned 0.68 more acres and accessed 0.63 more acres of land than their female counterparts. In terms of land ownership, there were no statistically significant gender differences. From the study, 44% of the farmers indicated that the land on which they farmed was jointly owned by the marital unit (man and woman). The remaining 56% reported a clear division of land ownership in the household with 30% stating that land was owned by adult man while 26% of the respondents reported that land was owned by the adult woman in the household. Traditional practices and customary laws often favor male inheritance and land tenure rights, limiting women's ability to own and control land. Additionally, women face challenges in asserting their land rights due to social norms, limited legal awareness, and inadequate implementation and enforcement of land laws that protect women's rights.

### Prices of common beans versus other crops

On the owned or rented piece of land, respondents grew various crops including maize, the staple, groundnut and potato. These crops were consumed in the home and sold in various markets. Compared to other crops, common bean fetched the highest price per kilogram (kg) on the market at the time of the study. Table 2 shows the common bean median price of MZN^2^ 25.00 (US$1.25) per kg, which was nearly four times that of the least paying crops, maize, and pigeon pea. This corresponds to the 2023 average wholesale price for maize^3^ which is between US$0.70 and US$1.40 per kg and common beans^4^ between US$2.1 and US$5.6 per kg.

**Table 2:** Median prices for various crops in Angonia district

Crop	Median price (MZN^a^ / kg)
Common bean	25 (US$1.25)
Groundnut	17 (US$0.85)
Soybean	10 (US$0.50)
Cowpea	10 (US$0.50)
Potato	8 (US$0.40)
Pigeon pea	7 (US$0.35)
Maize	7 (US$0.35)

*MZN* = *Mozambican Meticals, MZN1 is equivalent to US$0.05*

### What is the position of women and youth in the bean value chain?

There were differences across the age and sex categories in terms of bean production and marketing (Table 3). There was no significant difference in land under bean production among adult farmers. Unlike adults, land under beans differed significantly (p < 0.05) by sex among the youth. Young men’s bean production plots were 23% larger compared to young women’s 2.4-acre plots. These findings are nuanced as land ownership and control are often governed by customary laws and practices that tend to favor male inheritance and land tenure rights, resulting in a disproportionate distribution of land in favor of men, limiting women's access to and control over land. But there are instances where more equitable arrangements exist or where women have ownership more than men as this community is more matrilocal (Adam and Quinhentos 2018).

**Table 3:** Typology of bean production and marketing in Angonia district

Variable	Total (N = 332)	Young Men (n = 101)	Young Women (n = 44)	p-value	Adult Men (n = 119)	Adult Women (n = 68)	p-value
Land under beans (acres)	3.15	2.95	2.39	0.044	3.60	3.15	0.155
Source of bean seeds							
Own harvest	84.24	82.22	76.19	0.573	86.15	88.24	0.6136
NGO (free)	0.61	0	0.00		0.00	2.94	
Purchased	15.15	17.78	23.81		13.85	8.82	
Bean grain produce (kg)	519.70	501.51	407.39	0.790	576.25	515.55	0.858
Sold bean grain (Yes)	69.39	74.26	53.49	0.014	68.64	73.53	0.482
Bean grain sold (kg)	117.28	132.29	62.65	0.025	128.01	105.37	0.411
Income from beans (MZN)^a^	1952.14	2402.28	1074.71	0.004	2073.68	1557.87	0.002
Use of hired labour in bean production	41.87	40.59	36.36	0.6344	51.26	30.88	0.007
Access to credit (%)	11.75	12.87	6.82	0.288	15.97	5.88	0.044
Where grain was sold							
Farm gate	26.67	24.62	26.09	0.253	19.48	42.22	0.055
Local market	47.62	56.92	43.48		49.35	33.33	
Local town	14.29	13.85	13.04		16.88	11.11	
Distant town	11.43	4.62	17.39		14.29	13.33	
Transporting beans to market				0.004			0.4234
On foot	19.25	9.62	47.37		12.5	34.62	
Bicycle	57.76	65.38	42.11		64.06	38.46	
Ox cart	18.63	19.23	10.53		20.31	19.23	
Private vehicle	3.11	1.92	0.00		3.13	7.69	
Public transport	1.24	3.85	0.00		0.00	0.00	
Ownership of means of transport							
Bicycle	69.58	72.28	65.91	0.440	76.47	55.88	0.003
Ox cart	11.45	6.93	2.27	0.259	21.01	7.35	0.014
Vehicle	3.01	0.99	2.27	0.495	5.04	2.94	0.543

To establish the bean fields, the majority of the adult men, adult women, young men and young women reported using seeds saved from previous harvests. Additionally, adult men and young men produced more bean grain, 576 kg and 502 kg, respectively compared to adult women (516 kg) and young women (407 kg). The average plot size under common bean for each individual farmer was 3.15 acres. While there were no significant gender differences in the amount of bean grain produced by farmers, there were significant differences (p < 0.05) in the quantity of bean grain sold and the resultant income among the youth.

Market participation among the youth significantly differed by sex (p < 0.05). The amount of bean produce sold in the market differed by gender among the youth (p < 0.05), but not among adult farmers. Young men sold twice the volume of bean grain sold by young women. Young men sold an average of 132 kg while young women sold only 63 kg each. The young men earned the highest sales revenue across all the categories of farmers, which was 124% higher than that of young women. Similarly, adult men earned significantly (p < 0.01) more revenue at MZN2074.00 (US$103.70) than adult women at MZN1 558.00 (US$77.90). Furthermore, more young men (40%) than young women (36%) hired labour to work on bean fields, but the difference was not significant. Among adults, the differences were significant (p < 0.05); more adult men (51%) hired labour than adult women (31%).

The results show that overall, only 11.75% of the farmers had access to credit and there was a significant gender difference (p < 0.05) among adult men and adult women. The percentage (15.97%) of adult men farmers that had access to credit was more than twice that adult women farmers (6.88%). Similarly among the youth, more young men (12.87%) accessed credit than young women (6.82%), but the difference was not significant.

There was also a significant gender difference (p < 0.05) among young farmers regarding the means of transportation of harvested bean produce. More young men (65%) used bicycles to transport their produce compared to only 42% among young women. Moreover, while 2% and 4% of the young men used private vehicles and public transport respectively to transport their produce, none of the young women used these two means of transport. Similarly, none of the adult women used public transport. However, more adult women (8%) used vehicles for transport compared to adult men (3%). The results further demonstrated that there was a significant gender difference in bicycle ownership among adult farmers (p < 0.05). More adult men (76%) owned bicycles while only 56% of the adult women indicated owning bicycles. Among the youth, there was no significant gender difference in bicycle ownership. Young men (72%) and young women (70%) indicated owning bicycles and this was highly and positively correlated to the use of bicycles for transporting bean grain to the market (r = 0.68).

### Gendered points of sale and bargaining power

**Fig. 4:**
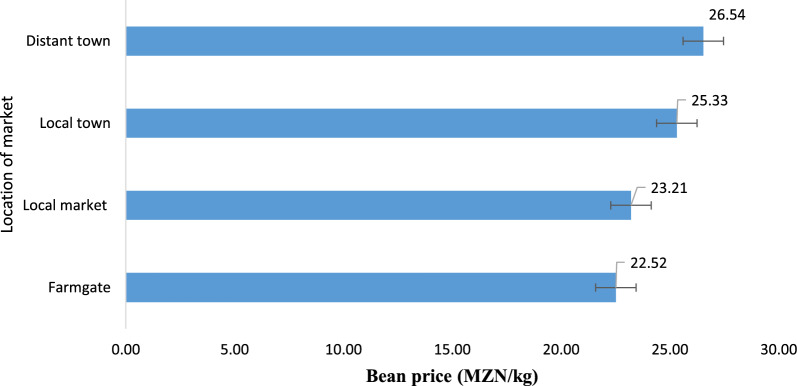
Bean prices (MZN per kg) by preferred point of sale (p < 0.01). *MZN* Mozambican Meticals*, MZN1* is equivalent to US$0.05

The results show that young men were more market-oriented than young women. A significant number of young men (74%) sold their produce to the market compared to young women (53%). The majority of the young men (57%) and young women (43%) sold their produce at the local market. The youth’s preferred points of sale did not differ by sex (p = 0.253). However, among adult farmers, the preferred points of sale differed by sex (p < 0.01). More adult men (49%) sold their produce at the local market, while the majority of the adult women (42%) sold their produce at the farm gate. As shown in Fig. 4, bean produce sold at the farm gate fetched the lowest price of MZN22.52 (US$1.13) per kg, followed by the local market at MZN23.21 (US$1.16) per kg, the local town at MZN25.33 (US$1.27) per kg, while distant town markets had the highest price of MZN26.54 (US$1.33) per kg.

Overall, there were more farmers owning bicycles (70%) when compared to the number of farmers owning other means of transport such as an ox cart (11%) while only 3% of the farmers indicated owning a vehicle. The results further demonstrated that there was a positive relationship (r = 0.68) between ownership and use of a bicycle for the transportation of bean produce to the market, suggesting that farmers who owned bicycles used them as means of transportation.

### Access and control over resources for common bean production

Table 4 presents the control of farm resources and decisions in the common bean farming enterprise as reported by men, women and youth farmers in Angonia district of Mozambique. The results further demonstrated the absence of significant gender differences in farmers’ responses concerning the control of bean production though more respondents (49%) reported that adult men controlled bean production compared to 26% who indicated dual control by both an adult man and adult woman in a household. In contrast, only 25% reported that adult women controlled bean production. Whereas there were no significant gender differences in the maker of the decision to plant the common bean among young men and young women, a significantly (p < 0.05) higher proportion of adult men (78.81) than adult women (50.75%) was responsible for the decision to plant beans. According to both young and adult men, women had the least sole decision; however, both young and adult women reported joint decision as the least common.

**Table 4:** Influence of gender on decisions and controls in the bean value chain

Variable	Total (N = 332)	Young men (n = 101)	Young women (n = 44)	p-value	Adult men (n = 119)	Adult women (n = 68)	p-value
Decision to grow beans				0.1632			0.051
Man	68.73	76.04	52.38		78.81	50.75	
Woman	13.62	4.17	30.95		2.54	35.82	
Both Man and Woman	17.65	19.79	16.67		18.64	13.43	
Who controls the sale of beans				0.621			0.718
Man	56.17	58.44	57.69		52.87	57.78	
Woman	15.32	11.69	23.08		17.24	13.33	
Both Man & Woman	28.51	29.87	19.23		29.89	28.89	
Control of bean revenue				0.9131			0.820
Man	54.89	58.44	53.85		51.72	55.56	
Woman	15.32	11.69	23.08		17.24	13.33	
Both Man & Woman	29.79	29.87	23.08		31.03	31.11	

Additionally, the majority of the young men and young women (58% each) and the majority of the men (53%) and women (58%) indicated that adult men controlled the sale of common bean grain (marketing) by a household. A significant majority of the farmers, adult men (52%), adult women (56%), young men 58% and young women 54% indicated that the adult man in the household had more control over the income generated from bean grain sales. This information is complemented by FGDs that stated that adult men were more interested in making decisions on income from bean sales.

Except for young women, all the other groups concurred on a pattern of the most control on the bean income, in descending order as follows; man, joint and woman. Young women reported equal proportions of men and women (23% apiece) controlling the income from bean sales.

Table 5 presents the results of a study that analyzed the typology of bean production and marketing among married and unmarried women bean farmers. The sample consists of 112 women, out of which 79 are married and 33 are unmarried. The table shows the mean values of three variables for married and unmarried women separately, as well as the p-values indicating whether there are statistically significant differences between the two groups. Married women produced slightly higher mean amount of bean grain (134.74 kg) compared to unmarried women (120.03 kg), but the difference was not statistically significant (p = 0.3249). Similarly, married women earned slightly, but not statistically significant (p = 0.4119) more income from beans (MZN1421.45 / US$71.07) compared to unmarried women who earned MZN1341.11 (US$67.06). By contrast, the percentage of women who have access to credit is significantly higher (p = 0.0293) among unmarried women (6%) compared to married women (0%).

**Table 5:** Typology of bean production and marketing among married and unmarried women bean farmers

	Total	Married women	Unmarried women	p-value
Variable	112	n = 79	n = 33	
Bean grain produced in kg		134.74	120.03	0.3249
Income from beans (MZN)		1421.46	1341.11	0.4119
Access to credit (%)	6	6	0	0.0293
Where grain bean is sold				0.7937
Farm gate	25	18	7	
Local market	26	20	6	
Local town	8	7	1	
Distant town	10	5	5	

*MZN* Mozambican Meticals*, MZN1* is equivalent to US$0.05

Lastly, the table also shows the distribution of where bean grain is sold among married and unmarried women. Most women sell their bean grain at the farm gate or in the local market, with a slightly higher percentage of married women selling at the farm gate compared to unmarried women. Overall, the table suggests that there are only minor differences in the typology of bean production and marketing between married and unmarried women, except for access to credit where unmarried women have a significant advantage.

Table 6 presents the availability of extension services and technical capacities reported by men, women and youth farmers in Angonia district of Mozambique. Agricultural extension systems were quite weak for legumes in Angonia. Less than seven percent of the respondents received legume-related extension services and there was no difference by sex. Only 10% of the respondents visited demonstration plots to learn about bean varieties and there were no gender differences across the young and old generations of farmers. The number of farmers with access to fertilizer remained relatively low at only 30% of the respondents. The majority of the farmers did not receive training on bean crop production (96%) or other crops (84%). Furthermore, 97% of the farmers also mentioned that they did not receive training in post-harvest handling of produce.

**Table 6:** Availability of extension and capacity development services

Variable	Total (N = 332)	Young men (n = 101)	Young women (n = 44)	p-value	Adult men (n = 119)	Adult women (n = 68)	p-value
Legume extension services (%)	6.63	3.96	6.82	0.464	10.08	4.41	0.171
Variety demonstration plot visit	9.64	9.90	6.82	0.554	10.08	10.29	0.964
Use of fertilizer in bean fields (%)	30.00	39.13	37.84	0.893	23.68	23.88	0.976
Information channel for grain prices							
Radio	30	23	22	0.871	24.00875	24.5	0.914
Talking to other farmers	148	53.446	53.20571	0.994	27.28951	35.33208	0.451
Talking to traders	23	20.15333	17.95	0.774	19.15333	17.95	0.551
Respondent paid for information				0.295			0.847
Yes	4.38	5.56	0.00		5.17	4.17	
No	95.62	94.44	100.00		94.83	95.83	
Respondent shared the information				0.009			0.159
Yes	85.51	91.67	63.16		84.75	95.83	
No	14.49	8.33	36.84		15.25	4.17	
Trained in bean crop production				0.457			0.152
Yes	4.22	4.95	2.27		5.88	1.47	
No	95.78	95.05	97.73		94.12	98.53	
Production training (other crops)				0.223			0.105
Yes (%)	15.36	16.83	9.09		19.33	10.29	
No (%)	84.64	83.17	90.91		80.67	89.71	
Post-harvest handling training				0.152			0.909
Yes (%)	3.31	1.98	2.27		5.88	1.47	
No (%)	96.69	98.02	97.73		94.12	98.53	
Grading or packaging training				0.169			0.682
Yes	2.62	2.67	4.35		3.70	0.00	
No	97.38	97.33	95.65		96.30	100.00	
Product processing training							
Yes	2.62	2.67	4.35		2.47	2.00	
No	97.38	97.33	95.65		97.53	98.00	

While there was not a significant gender difference among farmers who received grain legume extension services, there was a significant gender difference among adult farmers regarding extension services on cereals. More adult men (10%) than adult women (4%) received extension services on cereals. Farmers did not report significant gender differences in access to fertilizer; equal proportions of men and women (24% apiece) had access to fertilizer among adults, while the corresponding figures among the youth were 39% for young men and 38% for young women. Nonetheless, the overall number of farmers with access to fertilizer remained relatively low at only 30% of the respondents.

Regarding distance and price, the results in Table 7 demonstrated that the distance to the market influenced output prices. The correlation matrix shows a negative relationship between price and distance from the market; the lowest bean prices were in locations farthest from the market. Production locations farthest from the market may be inaccessible and as a result, they often suffer the double burden of the highest input costs and lowest commodity prices due to the absence of a competitive market. The results show that there was no significant gender difference in the mode of access to information. Among the communication channels relied on by farmers, the radio, talking to other farmers and talking to traders were classified as the primary sources of information on output prices.

**Table 7:** Bean price and distance to market correlation matrix

	Young women		Young men		Adult women		Adult men	
	Distance	Price	Distance	Price	Distance	Price	Distance	Price
Distance	1	− 0.1270	1	− 0.1776	1	− 0.1301	1	0.0100
Price	− 0.1270	1	− 0.1776	1	− 0.1301	1	0.0100	1

The results demonstrate that there was no significant price difference between adult men and adult women, and between young men and women regarding the communication channel they used to receive output prices. The majority of the farmers (96%) indicated that they did not pay for the market information that they received. However, there was a significant gender difference in information dissemination with more young men (92%) indicating that they share the information they received, while only 63% of the young women indicated that they shared the information they received. Among adult farmers, there was no significant gender difference regarding information sharing. Nonetheless, the number of adult women farmers who shared the information (95%) was slightly higher than the number of adult men (86%) doing the same.

## Discussion

We see a critical mass of literate farmers, especially women, which is an important factor for decision-making and closing some glaring gender gaps highlighted in this study. In terms of access to production resources, adult men accessed and owned more land than adult women. Access in this study is defined as a combination of owned, borrowed (free), or leased land (payable). In Mozambique, the land is owned by the state, but the land laws grant formal legal rights to customary rights and provide for private sector investment (Herrera Garibay et al. 2010) on unused land in agreement with surrounding communities (Hanlon 2011). Local communities and individuals have permanent occupation rights. In Angonia district, a predominantly matrilocal community, there should be almost equal ownership of land between the sexes. But this is not the case in the data as men own and access more land than women in both age categories. The results reveal gender inequality in land ownership and access, which may limit women decision making power on land use. Despite almost half of the respondents reporting joint ownership, the remaining proportion is skewed towards men's ownership, which may have implications for gendered division of labour, crops, and economic returns.

Our data shows that adult men and young men have more control over common bean production and proceeds from bean grain sales. Interestingly, adult men and adult women seem to downplay the control that the other sex has in the production and decision-making within the bean enterprise. This was reflected by the contrasting proportions of adult men and adult women that reported women’s autonomous control of bean production and sales. More adult women than adult men reported that adult women have control while more adult men than adult women reported joint control. By contrast, a study in Vanuatu, reported men’s undisputed control of decision making in agricultural activities; all participants, both adult men and adult women unanimously agreed that men were sole decision makers (Peralta 2022). Another study in Kenya also reported that adult men were in charge of decisions, more so for the lucrative export avocado crop compared to the domestic market (Oduol et al. 2017).

A female participant from Dzimeza village of Seze *Localidade* explained that ‘…*men get interested in beans when there is substantial revenue is generated; when we start growing the common bean, men are not interested, they even complain that the crop is distracting other farming activities through intercropping and additional labour. However, when they (men) realize that we (women) are getting good returns they get interested in knowing how much money we make, and the following season, they start controlling everything.’*

Validating the claim, another adult woman added that*, ‘we plant the common bean intercrops because men do not set aside land, time and labour for it, we therefore have to fit it somewhere with other crops.’* For men, the main argument was that* ‘Out of all the summer season crops, the common bean is the one that brings revenue earliest into the household, hence it is impossible for the man, the head of the household to lose track of early income.’*

In this study, FGDs highlighted that men’s control over revenue became more prominent as the size of the bean field increased or when there was a shift towards commercialization; otherwise, adult women generally have some control but not complete autonomy. Furthermore, FGDs revealed contrasting objectives on quantities of grain to keep for sale and to retain for seed for food.

An adult woman in FDG in Dzimeza village of Seze Localidade said *‘… men accuse us of desiring to keep everything for home consumption. Indeed, we are the ones struggling to provide the relish as our husbands tell us they have provided the main staple, therefore the sourcing of relish is on us, women.’* Highlighting the complexity in the sale of bean produce, one man also retorted,* ‘my wife accuses me of ‘stealing’ beans whenever I take bean grain to the market for sell; she feels the bean grain is her produce, but the field belongs to me.’*

Adult men have a propensity towards increasing the proportion of the grain produce sold, which in this study resulted in young men realizing more sales revenue, despite producing the same grain volume as their female counterparts. Similar to control of production, young women challenge the control of bean sales income by men; instead, they presume parity between joint control and adult women control. In contrast, adult women reported that adult women’s control of bean sales income as the least common control of bean sales income.

Adult men and young men produced more bean grains compared to their adult and young women counterpart. In addition young men compared to young women also sold more grains at better prices and this could be because adult men/young men have better market access, negotiating skills, or networks, that allow them sell more than adult/young women. Time and labour favour men and social norms like mobility restruct adult women access to better markets and prices (Me-Nsope and Larkins 2015; Siri et al. 2020).

Despite the various effort to close the gender gap in agriculture, evidence from this study still highlighted the inequalities faced by women in the bean value chain concerning control of resources, processes, and income in the absence of an intentional intervention to empower women and the youth. In other economic sectors, women still face hurdles in getting into leadership roles (Lunawat et al. 2021). Bean production has been traditional in Angonia, and investigation of this value chain offers an opportunity to understand the dynamics and help information interventions. This study presents the *status quo* of adult women and youth involvement in the bean value chain. It highlighted the shifts in women’s perspectives between the old and young generations, especially regarding the control of income.

In Mozambique common bean is still a woman’s crop but men are taking over the marketing and increasingly making decisions on what to sell and what to retain for food and seed. The result suggests possible change in gender roles in common bean farming. Regardless of age, women tended to be more involved in common bean production than men as shown by higher bean farming experience. This might be because women have valuable knowledge and skills in common bean production that could be useful in designing value chain interventions.

The bean crop is a preferred income-earner for farmers through competitive selling price per unit weight, which was the highest among all the crops produced in the district. Strengthening the crop's income potential may, however, require expanding the area under production and the productivity per unit of land area. Expanding the cultivation area requires more labour and/or mechanization to cope with the work. This is of particular importance to women, who in this study, hired less labour for common bean production when compared to men. Similarly, (Palacios-Lopez et al. 2017) reported a higher female share of labour in legume production in Malawi (53%), Tanzania (54%) and Uganda (59%), with the bulk of the labour going into primary operations such as land preparation, planting and harvesting.

## Conclusions

In this study, we found that young and adult men in Mozambique have greater access to land and control over common bean production and sales compared to young and adult women, despite the efforts to close the gender gap in agriculture. Both men and women tend to downplay the control that the other sex has in the production and decision-making process within the bean enterprise. Whereas adult women and young women had some control in bean production, they did not have complete autonomy.

Focus group discussions highlighted that young and adult men's control over common bean revenue was more prominent with larger bean fields compared to smaller bean fields. There were also contrasting objectives between adult men and young women regarding the quantities of grain to keep for sale, for seed and food. While adult and young women tended to keep their bean produce for home consumption, adult and young men sold their bean produce in the market.

Adult men and young men produced and sold more bean grains compared to their female counterparts, potentially due to better land ownership, better market access, enhanced negotiating skills, or networks. Time, labor, and social norms that restrict women’s mobility tend to limit their access to better markets and prices. The study highlighted the inequalities faced by adult women in the bean value chain, including control of resources, processes, and income, emphasizing the need for intentional interventions to empower young and adult women and youth.

Bean production, traditionally considered a woman's customary activity in Mozambique, is experiencing a shift as men increasingly take over marketing and decision-making regarding sales and retention of beans for food and seed. This suggests a possible change in the dynamics of common bean management and economic involvement in common bean farming by gender. However, women still tend to be more involved in common bean production than men, possibly due to their valuable knowledge and skills in this area. Both young and adult women hire less labor for common bean production compared to young and adult men.

Common bean is a preferred income-earning crop for farmers, offering competitive selling prices per unit weight. Strengthening the crop’s income potential may require expanding the cultivation area and improving productivity.

This study sheds light on the *status quo* of adult women and the youth involvement in the bean value chain, highlighting gender involvement in the bean value chain across generations, especially concerning benefit and control of income. It emphasizes the importance of understanding the dynamics of the value chain and implementing interventions to address gender inequalities. Furthermore, it underscores the need to empower women and overcome barriers that hinder their leadership roles in various economic sectors beyond agriculture. Policy interventions that address equal access to land, enhanced access to credit and gender sensitive training in agriculture are thus critical and can help improve young and adult women bean farmers control of and benefit from common bean production.

## Data Availability

The data is available from the authors upon request.

## Footnotes

1 Youth or young in this study is defined as 18 to 35 years as per the African Youth Charter. In Mozambique, the legal definition of "youth" is between the ages of 15 and 35 years.

2 MZN = Mozambican Meticals, MZN1 is equivalent to US$0.05.

3 https://www.selinawamucii.com/insights/prices/mozambique/maize/#:~:text=In%202023%2C%20the%20approximate%20wholesale,0.63%20per%20pound(lb).

4 https://www.selinawamucii.com/insights/prices/mozambique/beans/#:~:text=In%202023%2C%20the%20approximate%20wholesale,2.54%20per%20pound(lb).
